# The British Medical Association and the Voluntary Hospitals

**Published:** 1922-06

**Authors:** N. Bishop Harman

**Affiliations:** Chairman of the Hospitals Committee of the British Medical Association


### CORRESPONDENCE.

[Correspondence on all subjects is invited, but we cannot in any way be responsible for the opinions expressed by our correspondents, who must give name and address as a guarantee of good faith, but not necessarily for publication. Correspondents are reminded that conciseness greatly facilitates early insertion.]

### The British Medical Association and the Voluntary Hospitals.

*To the Editor of* THE HOSPITAL AND HEALTH REVIEW.

SIR,—I do not know if it be allowable to compliment an editor, but, if that be an orthodox procedure, I should like to congratulate you on the excellent summary you publish in the May issue of your journal on the Report of the British Medical Association on the Organisation of Voluntary Hospitals. I am also glad that you have noticed this report, for it is the work of many doctors, all of whom are keenly interested in our hospitals, and it is no mushroom growth or paper constitution, but the work of years. But in your few points of criticism I do not think you are so happy, if I may say so.

You criticise adversely four points of the scheme :—

1. The definition of a voluntary hospital.
2. The proposals for the remuneration of the staffs.
3. The scale of income limits for " tariff patients."
4. The suggestions for recommendation of patients.

1. The definition of a voluntary hospital. I think this is the first time that anyone has attempted seriously to define what is meant by a voluntary hospital. The definition was arrived at in 1920 at an open conference of hospital staffs. So soon as the subject was tackled, it was evident that support by gratuitous contributions and work by an unpaid staff did not define a voluntary hospital, for there are many which are supported by funds which are in the nature of payment for services rendered, and some by funds gotten by mass contributions, which are payments for services to be rendered. Also there are many hospitals where the honorary staff receive honoraria, and amongst these are to be counted some of the oldest and most celebrated. The one thing that differentiates our voluntary hospitals from State hospitals, from hospitals run by insurance companies (as in the U.S.A.), or by industrial firms in this country, is their independent management. When you come to consider the matter, I think you will agree that this is the essential feature of their success ; it secures their freedom to advance just as and when medicine shows powers of advance. That is the thing we wish to preserve. It is that " life and liberty " for hospitals which an established Church has sought to gain for itself in recent years.

2. Remuneration for the staffs of voluntary hospitals is an essential corollary to the reception of paying patients. In so far as patients pay, they cease to be the subjects of charity. It is no use pointing to the staffs of the few teaching hospitals of this country, some two dozen, and saying what a profitable office the membership of such a staff is indirectly. We have to deal with over a thousand voluntary hospitals, where there is much work done with little honour and glory, and no remuneration direct or indirect. That has to be remedied, otherwise the hospitals will suffer by lack of service. Already there has been difficulty in filling posts in some of the non-teaching hospitals. The difficulty of the situation is the most acute with the younger men. The loss of those patients who formerly came to the beginner because the hospitals have swallowed them up means that the beginner cannot live through the early years of his effort. The hospitals must see to the remedy of this difficulty in self interest. Some of them have done it. The B.M.A. scheme suggests how this can be done with even-handed justice, and without infringing any old charters defining the position of honorary staffs. The staff fund is a legitimate and sound proposition ; it has been proved.

3. The scale of income limit for the tariff patients has met with approval in other quarters than your own, for I see that a scale very like it has been adopted by the King Edward's Fund for London in its mass contribution scheme, so that, I take it, our scale is not so crude as you suggest. But you do not tell your readers that the report specifically states that the scale is " suggested subject to economic and local variations."

4. Your criticism is sharpest for the recommendation that " tariff patients " should only be admitted on the recommendation of a private practitioner, except in emergency. You write : " No one should be allowed to bar the patient's resort to the hospital." If you will forgive the statement, I think you have forgotten the conditions of a tariff patient. He is one for whom, in the main, someone has become responsible in conditions of illness which require " hospital benefit." The patient has provided beforehand by insurance or otherwise with some reputable authority or society for these benefits. It is therefore inevitable that the admission of the patient to hospital benefit must be subject to conditions, and the simplest of all is that he shall be recommended for admission to hospital by his own doctor, the one who has the care of him in his home. The absence of any such condition will mean the ruin of any contributory scheme, for you cannot always rely on the sound judgment of a patient as to what provision should be made for him. I do not think that you need have any fear that the doctor will unduly check the admission of patients to hospital, for it is equally certain that if a contributory scheme comes into successful operation there will be some form of appeal, indeed, this is provided for in our report by consultations for the patient, either at his own home or at the consultant's home. Finally, the recommendation is on all fours with that which makes the private doctor the arbiter of sickness benefit under the National Health Insurance Acts ; and I have no recollection that it has been charged against doctors that they have ever failed to certify patients when they were in need of such certification.

I trust, Sir, you will forgive the length of this letter. The subject is one of such importance, both to your readers as hospital managers, to the members of our profession, and to the public that there cannot be too much discussion of the report of the B.M.A.

I am,
Yours faithfully,
N. Bishop Harman,
*Chairman of the Hospitals Committee of the British Medical Association.*

---

[We are pleased to publish this letter, as it may be regarded as almost an official reply to our criticisms. We agree with our correspondent that discussion of the report is desirable, and, we would add, from other standpoints than that of a particular medical organisation. The future of the voluntary hospitals is a question in which the entire public, whether as subscribers or as beneficiaries, is deeply concerned. We do not think that our correspondent has seriously impugned any of our four criticisms. We proceed to deal with his answers.

(1) Though the term "voluntary hospital" may not have been defined before the conference of hospital staffs in 1920, its significance was perfectly understood as meaning an institution supported by voluntary subscriptions, not necessarily solely by them, for contributions towards maintenance are no new thing. Hospitals supported entirely by funds, which are in the nature of payment for services rendered, simply fall outside this definition and, therefore, their existence constitutes no objection. Voluntary management is a mere consequence of voluntary support, as we have pointed out The definition given by the Association is highly artificial, and is vitiated by the fact that it was made, not as a matter of lexicography, but in order to promote a particular policy.

(2) We do not agree that remuneration for the staff is a corollary to the reception of patients who pay anything. The essential point is whether the patient is a fit subject for the charity of the hospital or not. The out-patient department has been a feature of the voluntary hospital in the long period before "the general tendency to exact payment," which we are told fundamentally alters the basis of relationship between the honorary medical staffs and the subscribers. Yet no one suggested that because the out-patient was maintaining himself in his own home a contribution should be levied on him for the benefit of the medical staff. If under the new conditions he maintains himself in the hospital, the position is exactly similar. The hospital does not make a profit on the patient in which a share might be claimed. On the contrary, he usually costs it a good deal.

We disagree with our correspondent's limitation of the benefits from hospital appointments to the teaching hospitals. Our correspondent has confounded teaching hospitals with hospital schools. The figure he gives, two dozen, applies only to the hospitals with medical schools attached. The hospitals at which medical teaching is done must be about ten times that number. A hospital appointment of any kind is an advantage to the practitioner, and improves his status. Our correspondent makes a point of the difficulties of the beginner, which are only too real, but such difficulties are inevitable under any system. A man takes a hospital appointment with the object of acquiring the experience and skill which will give him a very different position from that of a general practitioner. He must devote years to acquiring these, during which patients who can pay consultants' fees will prefer to seek men of established reputation. The hospital work of the beginner is a kind of apprenticeship, of which the advantages are to be reaped later, and to those who cannot or will not undertake this struggle, general practice is open. The collection of a fraction of patients' payments for maintenance would have little effect on the position and would tend to obliterate the marked distinction between the consultant and the general practitioner, which is all to the advantage of the former, and, as is pointed out below, endanger the very system on which the status of the consultant depends.

(3) Our correspondent is wrong in stating that we did not tell our readers that the scale of income limit for tariff patients is suggested "subject to economic and local variations." We quoted these words in our summary. But this vague condition is no justification for the anomalies pointed out.

(4) Our correspondent's answer is quite irrelevant. The fact that the patient has provided beforehand, by insurance or otherwise, with some authority or society for hospital benefit in no way impairs his rights. We agree that as a general rule his doctor is the proper person to advise him, but that is a very different thing from depriving the patient of his right to apply for admission to hospital. The hospital authorities are the most competent persons to decide the question of admission, and cases certainly occur in which direct resort to them is wise.

To the fact, pointed out by the Cave Committee, that the carrying of a percentage of payments for the maintenance of patients to a staff fund would check subscriptions and endanger the whole voluntary system, our correspondent makes no answer. This view has been confirmed by members of the staffs of the large hospitals, and is shared by the great majority of the leaders of the medical profession.—Ed.]

